# Association between remnant cholesterol inflammation index and handgrip strength in maintenance hemodialysis patients

**DOI:** 10.3389/fendo.2026.1915243

**Published:** 2026-08-24

**Authors:** Qiao Yin, Jing Zhang, Xishuo Zhang

**Affiliations:** Department of Nephrology, The Second Affiliated Hospital of Shandong First Medical University, Taian, Shandong, China

**Keywords:** chronic kidney disease, handgrip strength, inflammation, maintenance hemodialysis, remnant cholesterol inflammation index

## Abstract

**Objective:**

This study examined the association between the remnant cholesterol inflammation index (RCII) and handgrip strength in patients receiving maintenance hemodialysis (MHD).

**Methods:**

This single-center cross-sectional study included 117 maintenance hemodialysis patients. Baseline characteristics, laboratory variables, and maximal handgrip strength were collected. Remnant cholesterol (RC) was calculated as total cholesterol minus LDL-C minus HDL-C, and RCII was calculated as [RC (mg/dL) × hs-CRP (mg/L)]/10. Because RCII was markedly right-skewed and included three verified negative calculated values, negative values were truncated to zero and ln(RCII + 1) was used as the primary exposure. The fully adjusted linear regression model included sex, age, BMI, creatinine, triglyceride, fasting plasma glucose, and albumin. Sensitivity analyses evaluated alternative treatments of negative and extreme RCII values, and restricted cubic spline analysis assessed potential non-linearity.

**Results:**

In the fully adjusted complete-case model (N = 116), each 1-unit increase in ln(RCII + 1) was associated with a 1.585-kg lower handgrip strength (B = -1.585, 95% CI -2.790 to -0.379; P = 0.010). The coefficient remained negative after excluding negative RCII values, using the original RCII scale, truncating negative values to zero, excluding extreme values, and fitting Huber robust regression. Statistical significance was attenuated after P1-P99 and 1.5×IQR exclusions. RCS analysis showed a significant overall association (P = 0.019) without evidence of non-linearity (P = 0.245).

**Conclusion:**

Higher ln(RCII + 1) was associated with lower handgrip strength in maintenance hemodialysis patients after adjustment for seven clinically relevant covariates. The inverse direction was broadly consistent across sensitivity analyses, although estimates were less precise after excluding upper-tail RCII observations.

## Graphical abstract

**Figure:**
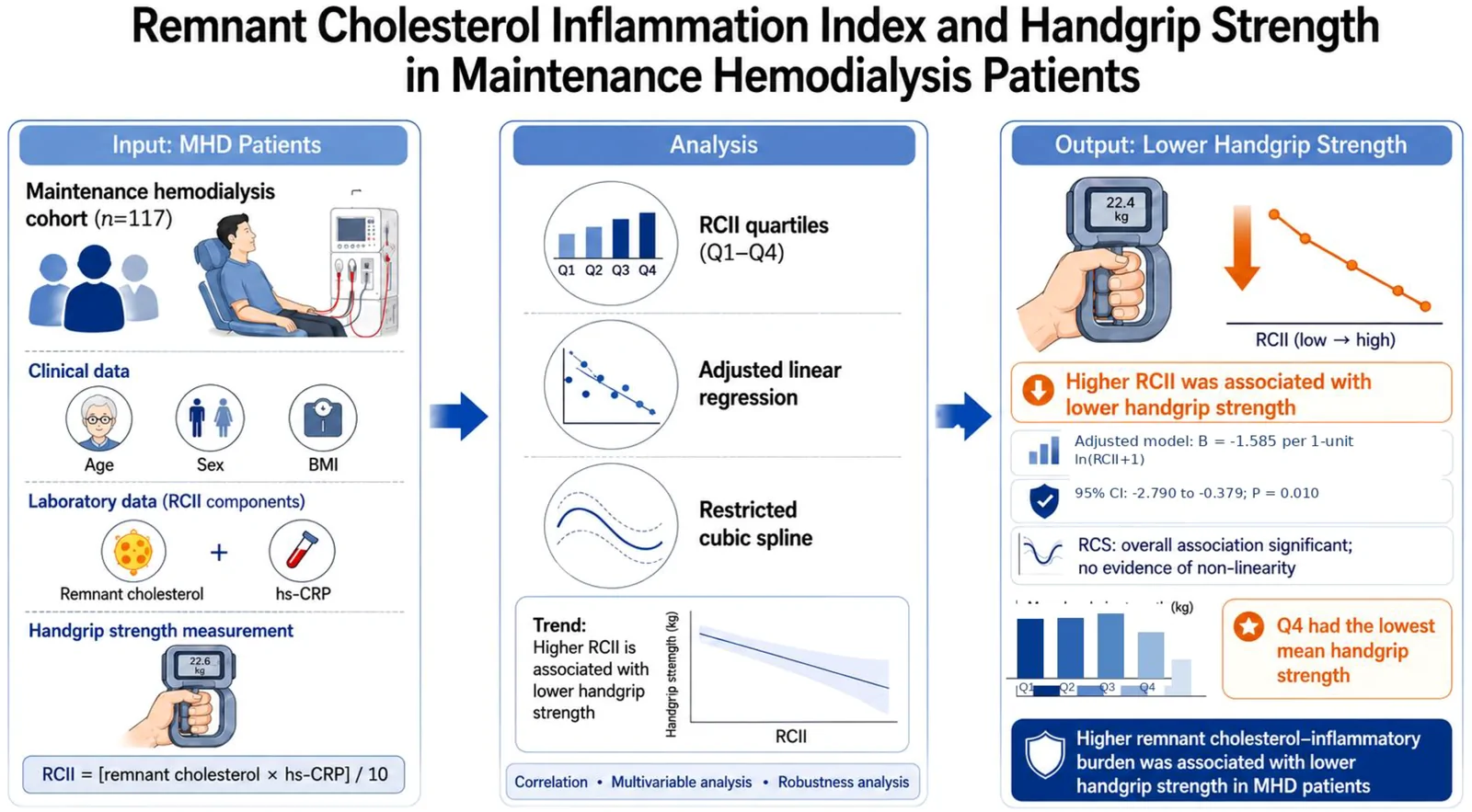

## 1 Introduction

Maintenance hemodialysis serves as a standard renal replacement therapy for stage 5 chronic kidney disease. At present, the survival rate of patients receiving maintenance hemodialysis has increased year by year. Sarcopenia is a clinical syndrome characterized by progressive loss of skeletal muscle mass and muscle strength (1).

The prevalence of sarcopenia ranges from 4% to 64% among patients undergoing hemodialysis (HD) (2). Sarcopenia severely impairs the clinical prognosis of hemodialysis patients (3, 4). Maintenance hemodialysis patients complicated with sarcopenia face elevated risks of cardiovascular and other adverse events, deteriorated quality of life, and even life-threatening outcomes (5).

Declined muscle strength constitutes one of the clinical manifestations and diagnostic criteria for sarcopenia. Isolated reduced handgrip strength (HGS) can indicate presarcopenia (6, 7).

Accumulating evidence links decreased muscle strength to unfavorable clinical endpoints in hemodialysis patients (8), rendering it a critical determinant affecting prognosis of maintenance hemodialysis recipients. Furthermore, handgrip strength has been identified as an independent correlate of poor health-related quality of life in MHD patients (9).

Remnant cholesterol (RC) refers to cholesterol carried by triglyceride-rich lipoproteins, encompassing intermediate-density lipoprotein cholesterol and very-low-density lipoprotein cholesterol in the fasting state, as well as chylomicron particles in the non-fasting state (10).

The remnant cholesterol inflammation index (RCII) is a composite metabolic-inflammatory biomarker integrating remnant cholesterol and high-sensitivity C-reactive protein. It may reflect the combined burden of dyslipidemia and chronic inflammation (11–15). Dyslipidemia and persistent inflammation are common in MHD populations, and heightened inflammatory status has been associated with lower skeletal muscle strength and mass (16–18).

Current research predominantly explores associations between RCII and cardiovascular, cerebrovascular, and metabolic outcomes. Direct investigations of RCII and muscle strength in MHD patients remain scarce. This study aimed to estimate the cross-sectional association between RCII and handgrip strength in MHD patients. It did not assess skeletal muscle mass or physical performance and therefore was not designed to diagnose or predict sarcopenia.

## 2 Subjects and methods

### 2.1 Study design and participants

Participants were consecutively enrolled from outpatients receiving maintenance hemodialysis at the Hemodialysis Unit of the Second Affiliated Hospital of Shandong First Medical University between November 2025 and May 2026. The study was a single-center cross-sectional observational study because RCII and handgrip strength were assessed during the same study period.

Inclusion criteria were: age ≥18 years; stable clinical condition without inpatient admission requirements; maintenance hemodialysis for ≥3 months; and a standard dialysis regimen of three sessions per week, 4 hours per session. Exclusion criteria were malignant tumor, tuberculosis or other chronic consumptive disorders; severe infection, active hemorrhage, or acute cardiovascular/cerebrovascular events within the preceding month; hepatic failure; congenital myasthenia; autoimmune disease or immunodeficiency; systemic severe infection; lower-limb paralysis or hemiplegia; severe arrhythmia; advanced cardiopulmonary insufficiency; psychiatric illness; or cognitive dysfunction. The participant selection flowchart is illustrated in **Figure S1**, which is provided in the Supplementary Material.

All patients underwent hemodialysis with high-flux membranes and standard bicarbonate dialysate. The study was approved by the Medical Ethics Committee of the Second Affiliated Hospital of Shandong First Medical University (No. 2025-H-0249), and written informed consent was obtained from all participants or their legal representatives.

### 2.2 Data collection

Demographic and clinical data included sex, age, pre-dialysis systolic and diastolic blood pressure, body weight, and body mass index (BMI). Fasting venous blood samples were collected before dialysis according to standardized laboratory procedures. Laboratory indicators included total cholesterol (TC), low-density lipoprotein cholesterol (LDL-C), high-density lipoprotein cholesterol (HDL-C), hs-CRP, fasting plasma glucose, albumin, hemoglobin, uric acid, creatinine, and other routine indicators.

Remnant cholesterol (RC) was calculated as TC - LDL-C - HDL-C. RCII was calculated as [RC (mg/dL) × hs-CRP (mg/L)]/10. This prespecified scale was used consistently in all tables, regression models, and figures.

LDL-C was directly measured using a homogeneous (direct) enzymatic assay and was not calculated by the Friedewald equation. Values were obtained from the routine laboratory information system and were not recalculated by the investigators.

Calculated RC and RCII values were checked against the original laboratory records and measurement units. Three participants (3/117, 2.6%) had negative calculated RC and RCII values because TC - LDL-C - HDL-C was below zero; no data-entry or unit-conversion errors were identified. These observations were retained in the primary analysis and addressed using dedicated sensitivity analyses.

### 2.3 Handgrip strength assessment

An M402858 electronic hand dynamometer (Haifuda Technology Co., Ltd., Beijing, China) was used to measure maximal handgrip strength on the non-fistula hand. Participants completed three maximal efforts separated by 5-minute rest intervals, and the maximum value was recorded. Handgrip strength was analyzed primarily as a continuous variable.

### 2.4 Statistical analysis

Statistical analyses were performed using SPSS version 26.0 and R software. Distributional assumptions for continuous variables were assessed using the Shapiro-Wilk test and visual inspection of histograms and Q-Q plots. Normally distributed variables are presented as mean ± standard deviation, skewed variables as median (interquartile range), and categorical variables as n (%). Missingness was reported for each variable. One creatinine value was missing (0.9%); analyses including creatinine used complete cases (N = 116).

Handgrip strength was approximately normally distributed. Differences in handgrip strength across RCII quartiles were evaluated using one-way analysis of variance. Because the quartile means were not monotonic, the quartile analysis was treated as descriptive; an exploratory linear trend test was additionally reported. Pearson correlation analysis was used only as an exploratory bivariate analysis and was not labeled as univariable linear regression.

The primary association between RCII and handgrip strength was estimated using multivariable linear regression. Because RCII was markedly right-skewed and included three verified negative calculated values, negative RCII values were truncated to zero and ln(RCII + 1) was used as the primary exposure. The fully adjusted model included seven prespecified covariates: sex, age, BMI, serum creatinine, triglyceride, fasting plasma glucose, and albumin. These covariates represented demographic characteristics, body composition/nutritional status, kidney function, and metabolic status. RCII components (RC, TC, LDL-C, HDL-C, and hs-CRP) were not entered simultaneously with RCII to avoid mathematical coupling and multicollinearity. Results are reported as unstandardized coefficients (B), standard errors, standardized coefficients (β), 95% confidence intervals, P values, adjusted R², and variance inflation factors. Analyses including creatinine used complete cases (N = 116).

Prespecified subgroup analyses were conducted by sex, age (<60 vs ≥60 years), and BMI (<24 vs ≥24 kg/m²). Subgroup models retained the remaining covariates, while the corresponding stratifying variable was omitted; interaction terms were used to assess effect modification. Sensitivity and robustness analyses included: (1) the primary fully adjusted ln(RCII + 1) model; (2) exclusion of the three negative RCII values; (3) analysis on the original RCII scale; (4) truncation of negative RCII values to zero on the original scale; (5) independent exclusion of observations outside the 1st-99th percentile ranges of handgrip strength or RCII; (6) independent exclusion using the 1.5×IQR criterion; and (7) Huber M-estimation robust linear regression without deleting observations. The P1-P99 and 1.5×IQR exclusions were applied independently to the full dataset. Restricted cubic spline analysis with four spline degrees of freedom was performed for ln(RCII + 1), adjusted for the same seven covariates; overall and nonlinear components were tested separately.

Dialysis vintage, Kt/V, vascular access type, residual kidney function, diabetes, cardiovascular disease, physical activity, medication use, and recent hospitalization were not collected in the present dataset and therefore could not be included in the adjusted model. Residual confounding by these variables is acknowledged.

## 3 Results

### 3.1 Baseline characteristics and data integrity

A total of 117 patients were included. Handgrip strength, age, sex, and the lipid and hs-CRP variables required to calculate RCII were complete for all participants. Serum creatinine was missing in one participant. Three participants (2.6%) had negative calculated RCII values; the values were retained in the primary dataset after verification against the source laboratory records. The baseline characteristics of the study population are shown in **Table 1**.

**Table 1:** Baseline characteristics of the study population.

Variable	N	Summary	Range
Sex, male/female	117	78 (66.7)/39 (33.3)	–
Age, years	117	56.0 (48.5, 63.0)	34.8-72.1 (P5-P95)
Weight, kg	117	68.90 (60.00, 77.20)	48.68-95.66 (P5-P95)
BMI, kg/m²	117	23.10 (21.15, 26.75)	18.45-33.15 (P5-P95)
Systolic blood pressure, mmHg	117	148.24 ± 20.36	100-195
Diastolic blood pressure, mmHg	117	87.37 ± 13.89	52-124
Urea, mmol/L	117	29.73 ± 8.24	13.25-51.57
Creatinine, μmol/L	116	1014.26 ± 300.10	315.1-1826.7
Uric acid, μmol/L	117	418.84 ± 117.48	117-859
Albumin, g/L	117	40.25 ± 3.22	30.9-47.9
Hemoglobin, g/L	117	112.56 ± 20.78	53-167
Total cholesterol, mmol/L	117	3.69 (2.94, 4.53)	2.43-5.82 (P5-P95)
Triglyceride, mmol/L	117	1.26 (0.85, 1.76)	0.51-3.26 (P5-P95)
LDL-C, mmol/L	117	1.94 (1.48, 2.48)	1.06-3.49 (P5-P95)
HDL-C, mmol/L	117	0.98 (0.81, 1.24)	0.60-1.77 (P5-P95)
hs-CRP, mg/L	117	3.15 (1.24, 7.21)	0.24-24.22 (P5-P95)
Fasting plasma glucose, mmol/L	117	5.15 (4.59, 6.64)	3.75-11.66 (P5-P95)
RCII	117	6.804 (3.128, 16.837)	0.361-79.263 (P5-P95)
Handgrip strength, kg	117	22.20 ± 8.70	7.3-46.4

Data are mean ± SD, median (P25, P75), or n (%), as appropriate. P5-P95 indicates the 5th to 95th percentile range. Creatinine was missing for one participant.

### 3.2 Handgrip strength across RCII quartiles

**Figure 1:**
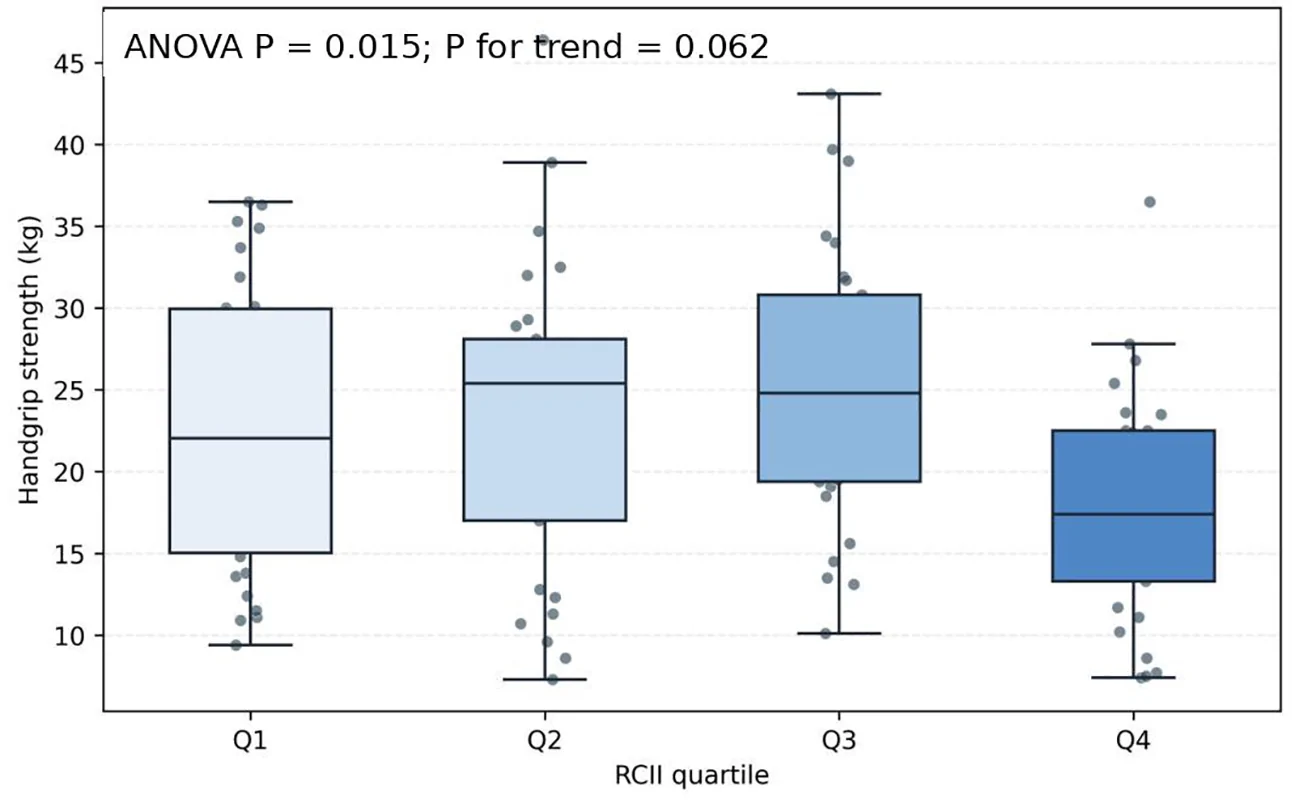
Handgrip strength across RCII quartiles (N = 117). One-way ANOVA: F = 3.643, P = 0.015; exploratory linear trend P = 0.062.

Handgrip strength differed significantly across RCII quartiles (one-way ANOVA P = 0.015), and Q4 had the lowest mean handgrip strength. However, the quartile means were not monotonic across Q1-Q4, and the exploratory linear trend did not reach conventional statistical significance (P for trend = 0.062). Accordingly, the main between-quartile difference was interpreted as being concentrated in the highest RCII quartile rather than as a progressive decline across all quartiles. **Table 2** and **Figure 1** present the handgrip strength distribution across RCII quartiles.

**Table 2:** Handgrip strength according to RCII quartiles.

RCII quartile	RCII range	N	Handgrip strength, kg
Q1	≤3.194	30	22.95 ± 8.60
Q2	>3.194-6.804	29	23.14 ± 9.40
Q3	>6.804-16.632	29	24.79 ± 8.49
Q4	>16.632	29	17.90 ± 6.99

One-way ANOVA: F = 3.643, P = 0.015. Exploratory linear trend: P = 0.062. Handgrip strength was approximately normally distributed (Shapiro-Wilk P = 0.078).

### 3.3 Exploratory Pearson correlation analysis

ln(RCII + 1) was inversely correlated with handgrip strength (r = -0.208, P = 0.025). This bivariate correlation was exploratory; the primary estimate was obtained from the fully adjusted multivariable linear model. The exploratory Pearson correlations with handgrip strength are listed in **Table 3**.

**Table 3:** Exploratory Pearson correlations with handgrip strength.

Variable	N	Pearson r	P value
Sex (male=1)	117	0.489	<0.001
Age, years	117	-0.397	<0.001
Weight, kg	117	0.394	<0.001
BMI, kg/m²	117	0.242	0.009
Creatinine, μmol/L	116	0.380	<0.001
Albumin, g/L	117	0.127	0.171
Total cholesterol, mmol/L	117	-0.297	0.001
Triglyceride, mmol/L	117	-0.181	0.051
LDL-C, mmol/L	117	-0.251	0.006
Hemoglobin, g/L	117	0.040	0.666
hs-CRP, mg/L	117	-0.103	0.271
Fasting plasma glucose, mmol/L	117	-0.123	0.187
TyG index	117	-0.181	0.051
RC index	117	-0.197	0.034
ln(RCII + 1)	117	-0.208	0.025

The analysis is presented as Pearson correlation rather than univariable linear regression. Correlations were exploratory and were not used as the sole basis for covariate selection.

### 3.4 Adjusted multivariable linear regression

After adjustment for seven clinically relevant covariates, higher ln(RCII + 1) remained independently associated with lower handgrip strength. Each 1-unit increase in ln(RCII + 1) was associated with an average 1.585-kg decrease in handgrip strength (B = -1.585, 95% CI -2.790 to -0.379; P = 0.010). Because the study was cross-sectional, this finding is interpreted as an adjusted association rather than a causal risk effect. **Table 4** presents the results of the fully adjusted multivariable linear regression model.

**Table 4:** Adjusted linear regression model for handgrip strength.

Variable	B	SE	Standardized β	95% CI	P value	VIF
ln(RCII + 1)	-1.585	0.608	-0.216	-2.790 to -0.379	0.010	1.371
Sex (male=1)	8.890	1.464	0.483	5.988 to 11.792	<0.001	1.262
Age, years	-0.214	0.060	-0.291	-0.333 to -0.094	0.001	1.353
BMI, kg/m²	0.206	0.160	0.101	-0.112 to 0.523	0.202	1.226
Creatinine, μmol/L	0.003	0.002	0.112	-0.002 to 0.008	0.182	1.399
Triglyceride, mmol/L	0.455	0.796	0.048	-1.123 to 2.033	0.569	1.383
Fasting plasma glucose, mmol/L	-0.129	0.194	-0.051	-0.513 to 0.256	0.509	1.192
Albumin, g/L	-0.010	0.206	-0.004	-0.419 to 0.399	0.962	1.166

Outcome: handgrip strength (kg). N = 116 complete cases. The model included ln(RCII + 1) and was adjusted for sex, age, BMI, creatinine, triglyceride, fasting plasma glucose, and albumin. Adjusted R² = 0.424; F = 11.590; model P < 0.001. All VIF values were <1.40, indicating no evidence of problematic multicollinearity.

### 3.5 Subgroup analysis

The estimated association between ln(RCII + 1) and handgrip strength was negative in all prespecified subgroups. None of the interaction tests was statistically significant, providing no evidence that sex, age, or BMI materially modified the association in this sample. Subgroup-specific significance was interpreted cautiously because of reduced precision and complete-case sample sizes within strata. **Table 5** displays the results of the subgroup analyses.

**Table 5:** Subgroup analysis of the RCII-handgrip association.

Subgroup	Level	N	B per 1-unit ln(RCII + 1)	95% CI	P value	P for interaction
Sex	Female	39	-1.488	-3.440 to 0.464	0.130	
Sex	Male	77	-1.795	-3.411 to -0.179	0.030	0.888
Age	<60 years	69	-1.825	-3.469 to -0.182	0.030	
Age	≥60 years	47	-1.491	-3.244 to 0.261	0.093	0.999
BMI	<24 kg/m²	65	-1.759	-3.213 to -0.305	0.019	
BMI	≥24 kg/m²	51	-1.059	-3.470 to 1.352	0.381	0.753

Subgroup models were adjusted for the remaining variables among sex, age, BMI, creatinine, triglyceride, fasting plasma glucose, and albumin; the stratifying variable was omitted from its corresponding subgroup model. Coefficients are expressed per 1-unit increase in ln(RCII + 1).

### 3.6 Sensitivity and robustness analyses

The RCII coefficient was negative in all seven analyses. The primary ln(RCII + 1) model, exclusion of the three negative values, analyses on the original RCII scale, truncation of negative values to zero, and Huber robust regression remained statistically significant. After P1-P99 and 1.5×IQR exclusions, the coefficients remained negative but were no longer statistically significant (P = 0.144 and P = 0.203, respectively). These findings support a generally consistent inverse direction but indicate reduced precision after excluding upper-tail RCII observations. **Table 6** summarizes the findings of the sensitivity and robustness analyses.

**Table 6:** Sensitivity and robustness analyses of the RCII-handgrip association.

Analysis	N	Exposure scale	B	Standardized β	95% CI	P value
Primary fully adjusted model	116	ln(RCII + 1)	-1.585	-0.216	-2.790 to -0.379	0.010
Exclude 3 negative RCII values	113	ln(RCII + 1)	-1.604	-0.209	-2.853 to -0.355	0.012
Original RCII scale	116	Per 10-unit RCII	-0.867	-0.237	-1.423 to -0.310	0.003
Negative RCII values truncated to 0	116	Per 10-unit RCII	-0.865	-0.236	-1.423 to -0.308	0.003
P1-P99 exclusion	108	ln(RCII + 1)	-0.926	-0.128	-2.171 to 0.320	0.144
1.5×IQR exclusion	99	ln(RCII + 1)	-1.083	-0.116	-2.761 to 0.595	0.203
Huber robust regression	116	ln(RCII + 1)	-1.741	-0.237	-2.903 to -0.580	0.003

All sensitivity models were adjusted for sex, age, BMI, creatinine, triglyceride, fasting plasma glucose, and albumin and therefore used complete cases. The P1-P99 and 1.5×IQR exclusions were applied independently to the full dataset rather than sequentially. Coefficients from ln(RCII + 1) models are expressed per 1-unit increase on the logarithmic scale and are not directly comparable with coefficients reported per 10-unit increase on the original RCII scale.

### 3.7 Restricted cubic spline analysis

**Figure 2:**
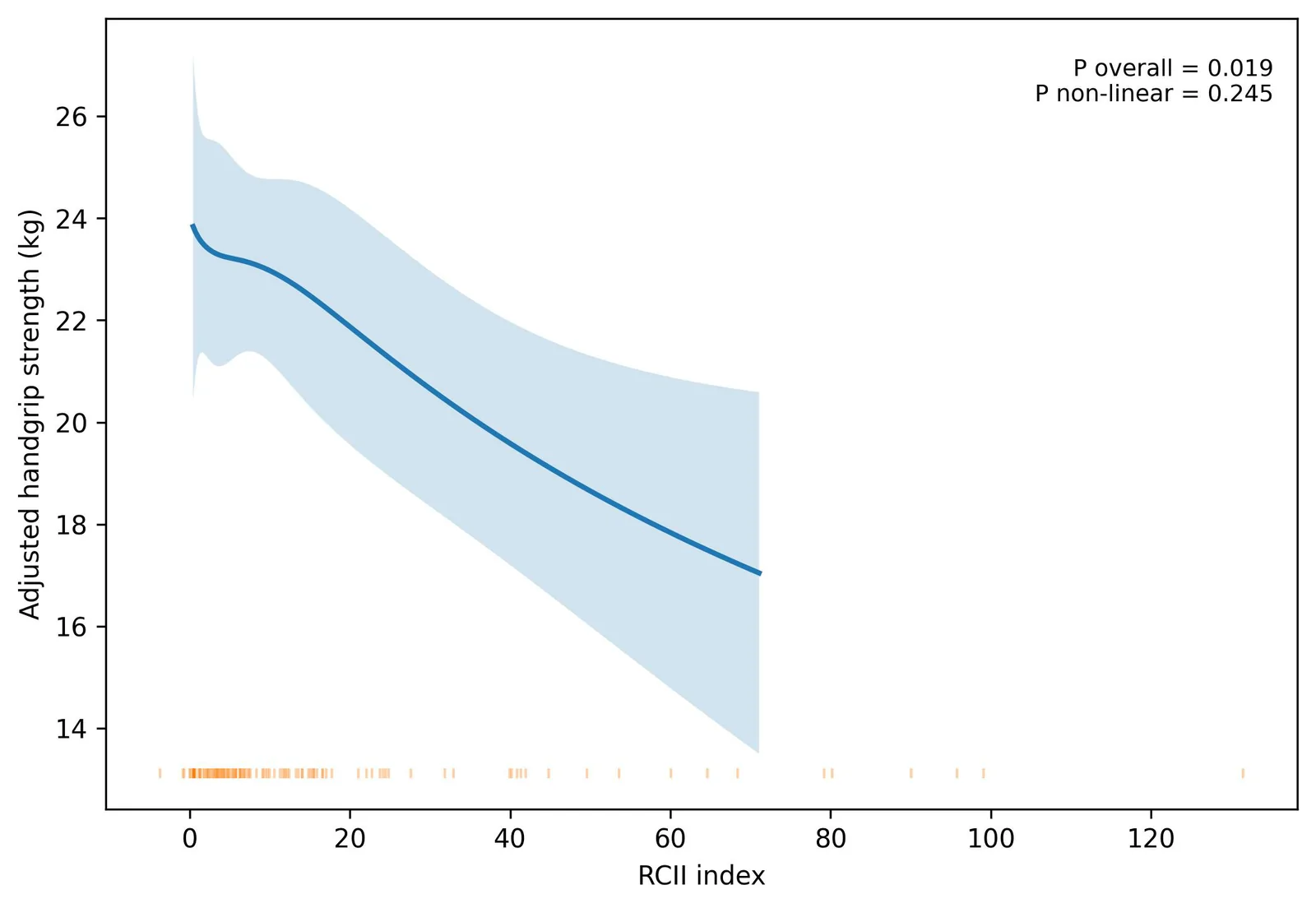
Restricted cubic spline for ln(RCII + 1) and adjusted handgrip strength (N = 116; adjusted for sex, age, BMI, creatinine, triglyceride, fasting plasma glucose, and albumin; four spline degrees of freedom; displayed over the 5th–95th percentile of RCII). Overall association P = 0.019; non-linear component P = 0.245. Shaded band = 95% CI.

The overall association between ln(RCII + 1) and handgrip strength was statistically significant (P = 0.019), whereas the nonlinear component was not (P = 0.245). The fitted restricted cubic spline curve is plotted in **Figure 2**. Thus, the data supported an approximately linear inverse association rather than a distinct threshold or U-shaped pattern. Confidence intervals widened at higher RCII levels because fewer observations were available in the upper tail. **Table 7** shows the results of the restricted cubic spline analysis.

**Table 7:** Restricted cubic spline analysis of RCII and handgrip strength.

Test	F	Df	P value
Overall association	3.097	4	0.019
Non-linear component	1.425	2	0.245

RCS analysis was adjusted for sex, age, BMI, creatinine, triglyceride, fasting plasma glucose, and albumin and modeled ln(RCII + 1) as the exposure. The spline basis used four degrees of freedom, and the curve was displayed over the 5th to 95th percentile range of RCII to reduce instability in sparsely populated tails.

## 4 Discussion

To our knowledge, this is among the first studies to examine RCII in relation to handgrip strength in MHD patients. Higher RCII was associated with lower handgrip strength after adjustment for measured covariates. Because the study was cross-sectional, RCII should be described as an associated factor or correlate rather than a risk factor, predictor, or causal driver. The study did not measure skeletal muscle mass or physical performance and therefore cannot support conclusions regarding sarcopenia diagnosis or prediction.

Chronic inflammation is common in MHD (19–22) and may contribute to impaired muscle protein synthesis (23–25), increased protein degradation (26, 27), oxidative stress (28), and reduced muscle function. Remnant cholesterol may also promote inflammatory and oxidative pathways (29, 30). These mechanisms provide biological plausibility for the observed association, but they were not directly measured in the present study and should not be interpreted as proven mediating pathways.

In the present dataset, RCII remained associated with handgrip strength in the clinically adjusted model, whereas several other markers did not. However, no formal statistical comparison of effect sizes or predictive performance among biomarkers was conducted; therefore, superiority of RCII over other biomarkers cannot be concluded.

The quartile analysis did not demonstrate a monotonic dose-response pattern across Q1-Q4; the lowest handgrip strength was concentrated in Q4. By contrast, the fully adjusted ln(RCII + 1) model and RCS analysis supported an overall inverse association. The negative direction was retained across all sensitivity and robustness analyses, including Huber regression; however, estimates became non-significant after P1-P99 and 1.5×IQR exclusions. Therefore, the findings should be interpreted as evidence of an inverse association that is partly sensitive to the upper tail of the RCII distribution, rather than as proof of a uniformly robust causal effect.

RCII is readily calculated from routine laboratory variables and may warrant further investigation as a metabolic-inflammatory correlate of handgrip strength. Clinical screening thresholds, risk prediction, and treatment decisions cannot be recommended from this cross-sectional analysis.

This study has several limitations. First, its single-center cross-sectional design precludes temporal and causal inference. Second, dialysis vintage, Kt/V, vascular access, residual kidney function, diabetes, cardiovascular disease, physical activity, medication use, and recent hospitalization were not available for adjustment, so residual confounding cannot be excluded. Third, calculated RC and RCII included three verified negative values and a markedly right-skewed upper tail. We used ln(RCII + 1) after truncation as the primary exposure and adjusted for seven available clinical covariates, but statistical significance was attenuated after excluding upper-tail observations. Larger multicenter studies with directly measured or rigorously harmonized lipid fractions and more complete dialysis-related covariates are required.

## 5 Conclusion

Higher ln(RCII + 1) was associated with lower handgrip strength in maintenance hemodialysis patients after adjustment for sex, age, BMI, creatinine, triglyceride, fasting plasma glucose, and albumin. The association was approximately linear and remained negative across multiple sensitivity analyses, but estimates were less precise after exclusion of upper-tail RCII observations. These findings support RCII as a potential metabolic-inflammatory correlate of muscle strength (31, 32) that requires confirmation in larger prospective cohorts (33, 34).

## Data Availability

The original contributions presented in the study are included in the article/**Supplementary Material**. Further inquiries can be directed to the corresponding author.

## Supplementary material

Supplementary Figure S1 Participant selection flowchart.
